# Influence of Cancerization of Lobules in Ductal Carcinoma In Situ of the Breast on the Pathological Outcomes in Mastectomy Specimens

**DOI:** 10.3390/cancers17101634

**Published:** 2025-05-12

**Authors:** Ferial Alloush, Hisham F. Bahmad, Arunima Deb, Stephanie Ocejo, Ann-Katrin Valencia, Amr Abulaban, Kritika Krishnamurthy, Sarah Alghamdi, Robert Poppiti

**Affiliations:** 1 The Arkadi M. Rywlin M.D. Department of Pathology and Laboratory Medicine, Mount Sinai Medical Center, Miami Beach, FL 33140, USA; ferial.alloush@msmc.com (F.A.); arunima.deb@msmc.com (A.D.); sarah.alghamdi@msmc.com (S.A.); robert.poppiti@msmc.com (R.P.); 2 Department of Pathology and Laboratory Medicine, University of Miami Miller School of Medicine, Miami, FL 33136, USA; amr.abulaban@jhsmiami.org; 3 Herbert Wertheim College of Medicine, Florida International University, Miami, FL 33199, USA; socej001@med.fiu.edu (S.O.); avale115@med.fiu.edu (A.-K.V.); 4 Department of Pathology, Montefiore Medical Center/Albert Einstein College of Medicine, Bronx, NY 10467, USA; kritika.myspace@gmail.com; 5 Department of Pathology, Herbert Wertheim College of Medicine, Florida International University, Miami, FL 33199, USA

**Keywords:** breast cancer, ductal carcinoma in situ, cancerization of lobules, extensive intraductal component, invasive ductal carcinoma

## Abstract

Cancerization of lobules (COL) is defined as the involvement of lobular acini by ductal carcinoma in situ (DCIS). Whether it represents a morphological variation in DCIS or a secondary extension of DCIS into lobules is debatable. The relation between COL and the probability of invasion is conflicting among different studies. We assessed if COL is a predictor of adverse pathological outcomes in mastectomy specimens. We reviewed the clinicopathological data of patients who underwent partial or total mastectomy for DCIS during a 3-year period (January 2015 until December 2017). Pathological parameters and follow-up data were collected. Whole-tissue hematoxylin and eosin (H&E) slides were reviewed and re-evaluated for COL. Cases with COL were stained immunohistochemically for E-cadherin and p120 to confirm the ductal phenotype of the neoplasms. In total, 171 mastectomies were identified including 65 specimens with pure DCIS and 106 specimens with DCIS with invasive carcinoma. COL was identified in 73 specimens. COL was significantly associated with adverse pathological outcomes including higher DCIS nuclear grade (*p*-value = 0.006), central (expansive “comedo”) necrosis (*p*-value = 0.008), presence of DCIS within or less than 2 mm from the surgical resection margin(s) (*p*-value = 0.004), higher percentage of blocks/slides with DCIS (*p*-value < 0.001), and extensive intraductal component (EIC) (applicable in cases with invasion) (*p*-value < 0.001). Invasion was seen in approximately two-thirds of the cases regardless of the presence of COL, with no statistical significance. Ninety-eight patients achieved 60 months of follow-up, of which only one patient developed local DCIS recurrence and had COL and EIC. Four other patients developed metastatic disease related to the invasive component. While other studies have previously hypothesized that COL may be associated with a worse pathological outcome at mastectomy, our results show that it may indeed be a measure of a higher disease burden representing EIC; however, it is not associated with an increased risk of detecting invasive carcinoma.

## Simple Summary

Cancerization of lobules (COL) occurs when the breast lobular acini are involved by ductal carcinoma in situ (DCIS), but its role in predicting invasive carcinoma remains unclear. Our study evaluated whether COL is linked to worse pathological outcomes in mastectomy specimens. We analyzed 171 cases of DCIS and found that COL is associated with factors indicating a higher disease burden, such as higher DCIS grade, central (“comedo”) necrosis, and extensive intraductal component (EIC). However, the presence of COL was not associated with an increased likelihood of detecting an invasive component. These findings suggest that COL may reflect a greater extent of disease but is not a direct predictor of invasion.

## 1. Introduction

Breast cancer remains the most frequently diagnosed cancer in women worldwide [1]. In the United States, and as of 2025, breast cancer alone accounted for 32% of the estimated new cancer cases among women [2]. The incidence rates of female breast cancer have been gradually rising by approximately 0.6% annually since the mid-2000s, mainly due to the increase in localized-stage and hormone receptor-positive cases [3]. In the last decade (2012–2019), the rise in breast cancer incidence has been more pronounced among women under 50 years of age (1.1% annually) compared to those aged 50 years and older (0.5% annually) [4].

Breast cancer encompasses a large and heterogeneous group of malignant epithelial neoplasms of the glandular elements of the breast. Those are distinguished by unique histological and biological characteristics, each exhibiting distinct clinical behavior and treatment responses. The World Health Organization (WHO) Classification of Tumors of the Breast is widely regarded as the gold standard for breast cancer diagnosis, as it provides internationally recognized criteria for classification [5]. Breast cancer can be categorized into subgroups based on histological features and tumor type, with the two primary types being ductal carcinoma and lobular carcinoma [6]. Invasive ductal carcinoma (IDC), also referred to as “invasive breast carcinoma (IBC) of no special type (NST)” by the WHO, accounts for approximately 65% to 85% of all breast cancers and is thought to originate from the milk ducts [6]. In contrast, invasive lobular carcinoma (ILC) comprises 5% to 15% of breast cancer cases and is believed to arise from the breast milk-producing lobules or glands of the breast [7,8,9,10].

Ductal carcinoma in situ (DCIS), also known as intraductal carcinoma, is a distinct non-invasive neoplastic entity characterized by the proliferation of cohesive epithelial cells confined within the mammary ductal–lobular system [5]. DCIS is typically a unifocal disease limited to a single ductal system; however, it may extend into the lobules, resulting in lobular involvement, a phenomenon referred to as lobular cancerization or cancerization of lobules (COLs). Given the unpredictable clinical course of DCIS, the standard management of DCIS often involves surgical excision—either lumpectomy or mastectomy—with or without adjuvant radiation and/or hormonal treatment [11].

**Figure 1:**
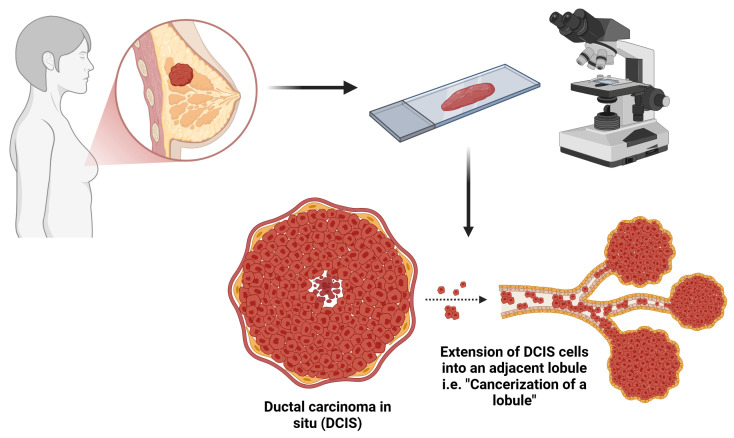
Schematic illustrating cancerization of lobules in ductal carcinoma in situ (DCIS). Created in BioRender. Bahmad, H. (2025) https://BioRender.com/n46n871 (accessed on 8 January 2025).

Cancerization of lobules (COL) refers to the extension of DCIS into the lobular units of the breast [12] (Figure 1). It was first described by Fechner in 1971 [12,13], and the term ‘cancerization of lobules’ was later introduced by Azzopardi in 1979 [14]. In his review of 205 breast carcinoma cases, Fechner emphasized that a carcinoma involving lobules is not necessarily indicative of lobular carcinoma. He identified 45 cases in which the tumor involved both terminal ducts and lobules, representing a pattern of ductal spread that should not be misclassified as lobular carcinoma in situ (LCIS) [12,13,15]. Even earlier, Foote and Stewart (1946) cautioned against confusing intralobular extension of intraductal tumors with LCIS [16]. Nevertheless, whether COL represents a morphological variant of DCIS or a secondary extension of DCIS into the lobules remains a subject of debate.

Lobular carcinoma in situ (LCIS) and COL exhibit distinct histopathologic features [12]. In LCIS, the lobular units are typically distended and globular in shape, with the acini completely filled by loosely cohesive tumor cells, leaving no residual lumina. The tumor cells in LCIS are generally bland, mitotically inactive, and lack a cribriform growth pattern or necrosis. LCIS commonly involves only portions of the ductal–lobular system. In contrast, COL usually involves lobular units of normal size, although occasional distention may be observed. Acini in COL retain luminal spaces, and the tumor cells tend to exhibit greater cohesiveness. Mitotic activity is comparable to that observed in ductal carcinoma, and tumor growth often demonstrates continuity from ducts into lobules. A cribriform growth pattern is often present, and necrosis may occur—either as focal (single cell) necrosis or as central (expansive “comedo”) necrosis. A prominent inflammatory and fibrotic stromal response is often observed around ducts involved by high nuclear grade DCIS, particularly when lobular involvement is present. Therefore, in such cases, it is crucial to thoroughly evaluate small, irregular cell clusters—rather than rounded ones—to exclude microinvasion, especially in the setting of a dense lymphocytic infiltrate [5,17]. Immunohistochemical staining for myoepithelial cell markers can always be used to assist in confirming the integrity of the myoepithelial layer surrounding involved lobules, though their assessment can still sometimes be challenging.

The association between COL and the likelihood of invasive carcinoma remains controversial across studies. In one study analyzing 200 cases of DCIS initially diagnosed on core needle biopsy and subsequently confirmed by surgical excision, an invasive carcinoma was identified in 21% of patients at the time of excision, with COL showing a significant association with invasion [18]. Similarly, another study of 91 cases of DCIS followed by excision reported that COL in lesions larger than 4 mm was significantly correlated with invasion [19]. In contrast, a study examining 157 cases of DCIS on biopsy found COL to be more prevalent in noninvasive cases (69%) compared to invasive cases (52%), suggesting a lack of association with invasion [20].

In this study, we present a retrospective cohort analysis to determine whether COL serves as an independent predictor of adverse pathological outcomes following mastectomy. The findings of this study may have clinical relevance in the preoperative risk stratification and treatment planning of patients diagnosed with DCIS with COL on needle biopsy.

## 2. Methods

### 2.1. Study Design and Setting

We conducted a single-center retrospective cohort analysis of breast cancer mastectomy specimens from patients treated at Mount Sinai Medical Center of Florida (Miami Beach, FL, USA) over a three-year period, from 1 January 2015, to 31 December 2017. Pathology reports were reviewed using “CoPath” (Sunquest information Systems, inc., Tucson, AZ 85718, USA), the institution’s computerized pathology database. A comprehensive chart review was performed to collect patient information, ensuring no direct physical risk to participants in the study.

### 2.2. Ethical Considerations

Approval of the Institutional Review Board (IRB) of Mount Sinai Medical Center of Florida was granted prior to commencement of the study (FWA00000176; 5 April 2023). All protocols followed in our retrospective cohort study were performed in accordance with guidelines and regulations of The Code of Ethics of the World Medical Association (Declaration of Helsinki). The study was performed in a manner that ensures confidentiality of patients. The chart review was carried out by CITI (Collaborative Institutional Training Initiative) certified resident physicians. All data collected were de-identified and stored at the principal investigator’s office.

### 2.3. Patients’ Selection

Inclusion criteria included treatment-naïve patients, aged less than 90 years old, who underwent mastectomies for DCIS with or without invasion during the time period specified. Exclusion criteria included patients who have received any form of chemo- or radiation therapy or neoadjuvant therapy prior to surgery.

In our study, we obtained a list of the pathological case numbers from the database search results and pulled out all slides of patients who met the inclusion criteria. Two pathologists (R.P. and S.A.) re-reviewed the diagnoses made previously by multiple pathologists. Henceforth, no cases had to be excluded because of a disagreement with the pathologist’s diagnosis. The diagnosis of DCIS, with or without COL, was made by several pathologists based on morphologic and Hematoxylin and Eosin (H&E) examination of tumor tissues where pathologists made a diagnosis when there was unequivocal pathologic evidence of intraductal carcinoma. To support the ductal phenotype of the “in situ carcinoma”, we performed immunohistochemical stains for E-cadherin and p120 on all cases and confirmed the presence of strong membranous immunopositivity. Two pathologists (R.P. and S.A.) re-reviewed and re-evaluated the slides for COL, number of blocks/slides with DCIS total number of blocks examined, and other histopathological parameters such as necrosis, grade, stage, margin status, presence or absence of extensive intraductal component, and invasion, among others.

### 2.4. Clinicopathological Variables of Patients

Clinical and pathological data of patients were retrospectively retrieved from electronic medical records, including age, history of adjuvant or neoadjuvant therapy, and the mastectomy pathological variables: procedure type, histologic type of invasive carcinoma (if applicable), histologic grade of invasive carcinoma (if applicable), tumor size (if applicable), tumor focality, presence of extensive intraductal component (EIC; if applicable), number of blocks with DCIS number of blocks submitted for microscopic examination, presence of cancerization of lobules (COL), number of blocks with cancerization of lobules (if applicable), architectural patterns of DCIS, nuclear grade of DCIS, presence of necrosis and type of necrosis in DCIS, extent of invasive carcinoma (if applicable), surgical margins status, regional lymph node status, distant metastasis, and pTNM stage (AJCC 8th Edition). In addition, follow-up data including recurrence and survival status were recorded.

### 2.5. Histopathologic and Immunohistochemical Staining and Evaluation

**Figure 2:**
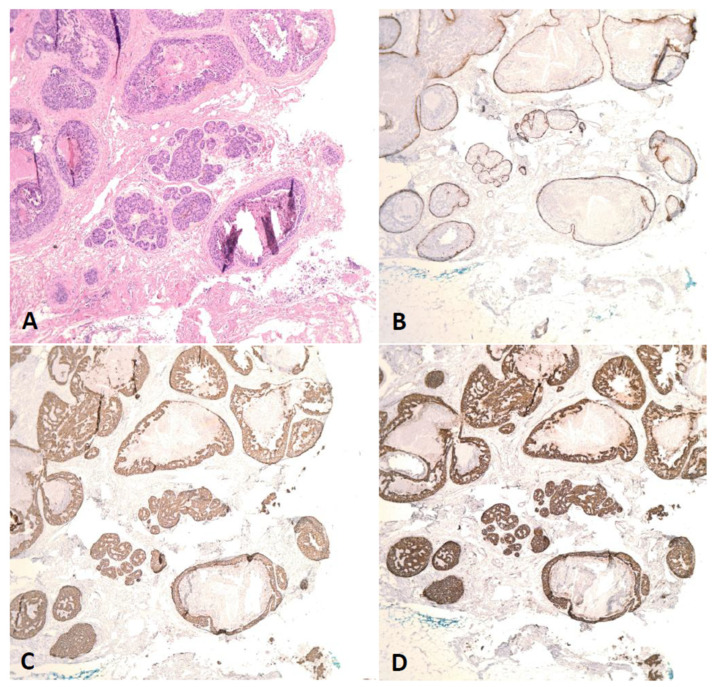
DCIS, intermediate nuclear grade, solid type, with cancerization of lobules (COL). (**A**) Solid DCIS with involvement of an adjacent lobule (H&E, 50×). (**B**) Retained myoepithelial cells around the lobules are highlighted by calponin immunohistochemical staining (50×). (**C**) Circumferential membranous immunoreactivity for E-cadherin supporting ductal phenotype (50×). (**D**) Circumferential membranous immunoreactivity for p120 supporting ductal phenotype (50×).

Formalin-fixed, paraffin-embedded tissue samples slides were stained with E-cadherin and p120 immunohistochemistry (IHC). The corresponding Hematoxylin and Eosin (H&E) slides were analyzed in accordance with their compatible IHC stains. IHC staining for E-cadherin (clone 36, Cell Marque company, Rocklin, CA, USA) and p120 (clone MRQ5, Cell Marque company) was performed in sections cut at 4 µm from formalin-fixed, paraffin-embedded (FFPE) tissue blocks and using the automated IHC protocols by Ventana Medical Systems. Appropriate positive and negative controls were used. Screening of the tumor tissues was performed in a systematic manner. Ductal phenotype was confirmed when the “in situ” neoplastic cells show retained circumferential membranous immunoreactivity for both E-cadherin and p120 (Figure 2).

### 2.6. Statistical Analysis

Data retrieved from the electronic medical records and pathology reports of patients were entered into a Microsoft Excel spreadsheet which was designed specifically for this study. Statistical analyses, data management, and cleaning were executed using the SPSS (IBM Corp., Released 2019, SPSS Statistics for Windows Version 26.0, Armonk, NY, USA). Descriptive statistics were reported as frequencies and percentages for categorical variables and means (±) standard deviation for continuous variables. Pearson’s Chi-squared χ^2^ test was used to assess the association of the COL status categorized into two groups (COL-YES and COL-NO) with the patients’ clinicopathological characteristics. Univariate Cox regression analyses were used to assess the influence of COL on tumor stage, lymph node status, microinvasion, surgical margin status for DCIS, percentage of tumor blocks/slides with DCIS, presence of focal or comedo-type necrosis, presence of extensive intraductal component (EIC), and nuclear grade of DCIS. Variables found to be significant in univariate analyses were subjected to multivariate analysis. *p* values less than 0.05 were considered statistically significant.

## 3. Results

Our final cohort included 164 treatment naïve patients who underwent mastectomy for DCIS. Among those, 171 mastectomy specimens were identified, including 111 partial mastectomies (lumpectomies) and 60 total mastectomy specimens. Seven patients had synchronous or metachronous diseases affecting the contralateral breast. The patients were 161 women and 3 men. A total of 65 specimens showed pure DCIS (without invasion), and 106 specimens showed DCIS with invasive carcinoma. The age at the time of diagnosis ranged from 28 years to 91 years with an average of 63.5 years. COL was identified in 73 specimens (Table 1).

**Figure 3:**
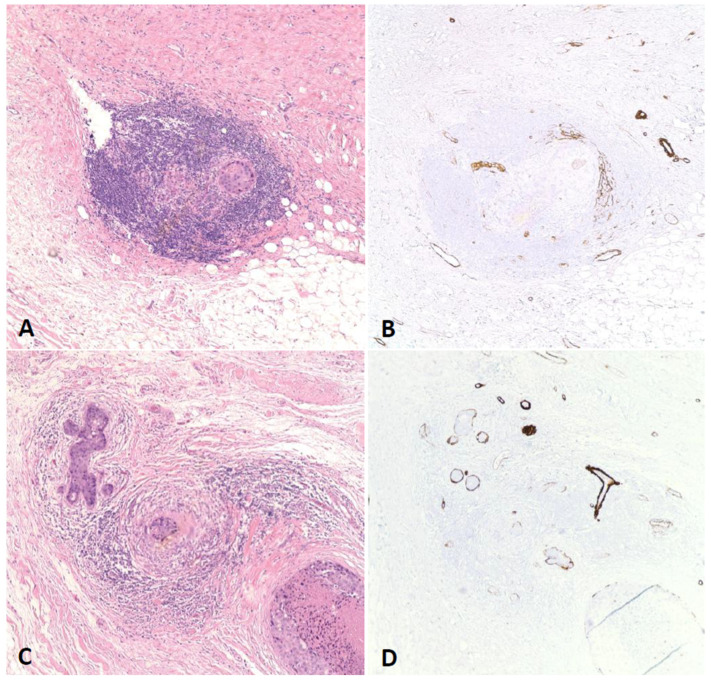
(**A**). A focus of microinvasion (0.5 mm) with lobulated contours mimicking COL (100×). (**B**). Loss of myoepithelial cells in the focus of microinvasion highlighted by negative smooth muscle myosin immunohistochemical staining (100×). (**C**). DCIS with COL found in adjacent breast tissue (100×). (**D**). Intact myoepithelial cells in the focus of DCIS are highlighted by positive smooth muscle myosin immunohistochemical stain (100×).

COL was significantly associated with several adverse pathological outcomes including higher DCIS grade (*p*-value = 0.006), comedo necrosis (*p*-value = 0.008), presence of DCIS within 2 mm of surgical margins (*p*-value = 0.004), a higher percentage of blocks/slides with DCIS (*p*-value < 0.001) and presence of EIC (only applicable in cases with invasion) (*p*-value < 0.001). Invasion was seen in approximately two thirds of the cases regardless of the presence of COL, with no statistical significance (Table 1; Figure 2 and Figure 3). Univariate regression analyses showed similar results (Table 2). Under multivariate analysis, COL was no longer significantly associated with margin status of DCIS (*p*-value = 0.53) or comedo necrosis (*p*-value = 0.17); however, it showed significant independent predictability for higher DCIS grade (*p*-value = 0.01), higher percentage of blocks/slides with DCIS (*p*-value < 0.001), and presence of EIC (*p*-value = 0.002, OR = 9.28) (Table 3). In addition, percentage of blocks/slides with DCIS, presence of EIC, and DCIS grade all showed reciprocal independent predictability for presence of COL (Table 4).

Cribriform DCIS was significantly associated with higher DCIS grade. Comedo-type DCIS showed a significant association with both higher grade and a greater percentage of blocks containing DCIS. Similarly, micropapillary DCIS was significantly associated with an increased percentage of blocks involved by DCIS (Table 3). Ninety-eight patients achieved 60 months of follow-up, of which only one patient developed local DCIS recurrence. COL and EIC were present. Four other patients developed metastatic disease related to the invasive component. Three deaths were recorded within the patient cohort. Two of them were attributed to metastatic breast cancer, while one was related to chronic kidney disease.

## 4. Discussion

Different studies have reported that 25% to 60% of untreated DCIS progresses to invasive ductal carcinoma within 9 to 30 years of follow-up [21,22,23,24]. Differentiating indolent DCIS from DCIS that is prone to progression is essential in preventing undertreatment or overtreatment. Yet, the natural history of this progression is still poorly understood. Synchronous DCIS and invasive ductal carcinoma is a frequent finding. Multiple studies have concluded the high levels of genetic concordance between DCIS and invasive ductal carcinoma [25,26,27,28]. Thus, it has been inferred that the majority of DCIS found synchronously with invasive ductal carcinoma represents the precursor lesion that has undergone the genetic changes necessary for the progression of DCIS into invasive ductal carcinoma [29].

Huo et al. reviewed 200 patients who had DCIS on initial core needle biopsy followed by surgical excision. Invasive carcinoma was identified in 41 patients (21%) on final excision. Lobular cancerization was found to be associated with invasion (Odds ratio: 2.70, 95% CI, 1.27–5.74). The difference was statistically significant on univariate as well as multivariate analysis [18]. Renshaw et al. found in their study of 91 cases of DCIS on biopsy with subsequent excision, that cancerization of lobules in lesions greater than 4 mm is associated with invasion (*p* = 0.03) [19]. On the contrary, Go et al. studied 157 biopsies with DCIS. Invasive carcinoma was identified in 48 cases on subsequent surgical excision. Cancerization of lobules was significantly more common in the noninvasive group (69%) when compared with the invasive group (52%) (*p* = 0.045) [20]. In our study, concurrent invasion was seen in approximately 60% of cases of DCIS with cancerization of lobules compared to approximately 64% of cases of DCIS without cancerization of lobules, thus cancerization of lobules was not associated with a higher risk of invasion. A potential limitation of is the low interobserver agreement among pathologists regarding COL. In an international study that included dichotomous evaluation of 149 consecutive DCIS cases by 39 pathologists, the Krippendorff’s alpha (KA) value was found to be 0.396 [30]. Interestingly, interobserver agreement was higher among pathologists that universally report the presence of COL in their cases, when observed. To address this and ensure that cases with unreported but present COL were not missed, all slides from selected cases were re-reviewed. This review was first conducted by a resident involved in the project and subsequently confirmed by an attending pathologist. Only cases where there was consensus on the presence of COL were categorized as COL-present.

Other clinicopathological factors associated with invasion include lesion size greater than 1.5 cm and mass lesion rather than calcifications on imaging [18]. Some studies have concluded that higher nuclear grade and the presence of comedo necrosis were predictors of invasion [31,32,33], but not others [34,35]. Similarly, some studies have found that periductal chronic inflammation is associated with higher risk of invasion [33], but not others [18]. The role of p16 overexpression has also been explored. According to Rohan et al. [36], p16 overexpression in DCIS was not linked to an increased risk of developing ipsilateral invasive breast carcinoma. In contrast, the study by Shan et al. found that p16 overexpression was strongly associated with poorer outcomes and progression to more advanced disease in luminal A and luminal B DCIS subtypes [37]. However, this correlation was not observed in invasive ductal carcinoma, suggesting that p16 may play a role in the early stages of tumorigenesis, but its impact may be diminished or obscured by other genetic alterations as the disease progresses.

Tumors classified as EIC are those that contain a minimum of 25% ductal carcinoma in situ (DCIS) component and extend to the surrounding normal breast tissue [38]. It is present in approximately 24–35% of breast cancer patients [39,40,41]. Polchai et al. found in their metanalysis of 32 studies, comprising 19433 patients that EIC is associated with a higher risk of local recurrence (OR = 2.73; 95% CI: 2.42–3.07; *p* < 0.00001). However, the difference was not statistically significant in patients who had negative margins or received adjuvant radiation [38]. Interestingly, the relationship between COL and EIC has not been explored. In our study, we found cancerization of lobules to be significantly associated with EIC on univariate and multivariate analysis, with the presence of EIC in approximately 65% of cases where COL was present compared to approximately 14% of cases without COL. However, a significant limitation of the concept of EIC was that it can only be measured in cases where an invasive component is present. Hence, we recorded the number of slides/blocks with DCIS and the overall number of blocks submitted from a partial/total mastectomy specimen to measure disease extent in all cases. The percentage of blocks involved with DCIS was greater than 30% in approximately 61% of cases with COL compared to approximately 15% of cases without COL. These findings suggest that COL is associated with a significantly higher DCIS burden. Thus, we hypothesize that COL represents a morphological variation in DCIS, indicative of a subset of the disease with a tendency for extensive growth within the ducts, yet a relatively low propensity for invasion.

In our study, COL was associated with comedo-type necrosis (OR: 1.65, *p*-value: 0.002). A possible explanation might be that comedo necrosis is characterized by central necrosis within ducts due to rapid tumor cell proliferation outpacing its blood supply. Similarly, COL is characterized by increased cellular proliferation resulting in the extension of DCIS beyond the small ducts into the lobular unit. This was reflected in our results, with COL being associated with increased disease burden.

In our study, COL was significantly associated with a higher risk of the presence of DCIS with 2 mm of surgical resection margin on univariate but not on multivariate analysis. One plausible explanation is the presence of confounding variables—such as tumor size, grade, or extent of disease—that may account for the observed association in unadjusted analyses. Additionally, given the relatively small sample size, our study may not have adequate power to detect a true independent effect. Furthermore, institutional surgical practices may have influenced our findings. At our institution, it is routine to take additional tissue samples from the lumpectomy cavity edges, which are reported as final margins. Additionally, intraoperative frozen section evaluation is commonly employed. These practices may contribute to a lower frequency of positive margins, potentially limiting the generalizability of our results to centers with different surgical protocols.

The relationship between COL and margin status is not extensively studied in the literature. However, a 1998 study [42] involving 94 patients who underwent lumpectomy followed by radiation, with a median follow-up of 78 months, found that nine patients developed local recurrence. It revealed a significant association between high-grade DCIS and COL within 2 mm of the surgical margin, with recurrence and poor radiation response. This may be due to the fact that, at the time, a 2 mm clear margin was not yet the standard of care in DCIS cases. In 2016, a consensus statement was issued by the Society of Surgical Oncology, the American Society for Radiation Oncology, and the American Society of Clinical Oncology, concluding that a negative margin of ≥2 mm reduced the risk of local recurrence [43]. Further studies with larger sample sizes may help clarify the independent role of COL in predicting surgical margin outcomes and deciphering the potential role of adjuvant radiation in DCIS with COL.

Adjuvant radiation is the standard of care following breast-conserving surgery in women with isolated DCIS due to a relative risk reduction in local recurrence of approximately 50% with the addition of adjuvant radiation [44,45,46,47,48]. However, there is increased concern in recent years regarding overtreatment of DCIS given the indolent course of the disease, delayed recurrence and the absence of survival benefit. Efforts are now focused on identifying patients with low-risk DCIS, for whom the risks of radiation may outweigh the potential benefits [49]. Our findings suggest that COL does not appear to be associated with high-risk disease. Notably, the existing literature tends to focus on the risk of DCIS recurrence as a negative clinical outcome. We propose that future studies should prioritize examining the risk of invasive disease recurrence, as this is more likely to impact survival outcomes.

While our findings do not yet warrant a shift in surgical protocols, the increased disease burden linked to COL implies that achieving negative margins may be more difficult. Therefore, it could be beneficial to consider additional measures, such as obtaining extra tissue from the lumpectomy cavity and/or conducting intraoperative gross and frozen section evaluations of margins. These approaches might help ensure clear margins and better outcomes for patients with COL.

A limitation of our study is the relatively short follow-up period, which limits our ability to assess the potential impact of COL on recurrence and survival. Another limitation is the relatively small sample size, which may affect the generalizability of our findings. Due to the commonly protracted course of DCIS, we suggest conducting cohort studies with extended follow-up periods (e.g., 10–20 years) to capture data on recurrence and survival outcomes in patients with and without COL.

## 5. Conclusions

In conclusion, while other studies have previously hypothesized that COL may be associated with a worse pathological outcome at mastectomy, it appears to us that it is, indeed, a measure of a higher disease burden representing EIC; however, it is not associated with an increased risk of detecting invasion. Further studies are necessary to evaluate recurrence risk in DCIS with COL and elucidate the necessity for adjuvant radiation in patients with COL.

## Figures and Tables

**Table 1:** Clinicopathological features of DCIS cases stratified between those with COL (COL-YES) vs. those without COL (COL-NO).

Clinicopathological Features	Total Patients	COL-NO	COL-YES	*p*-Value
EIC (n = 106)				
No	69	54 (85.7%)	15 (34.9%)	<0.001 *
Yes	37	9(14.3%)	28 (65.1%)	
% of blocks/slides with DCIS (n = 171)				
≤30%	111	83 (84.7%)	28 (38.4%)	<0.001 *
>30%	60	15 (15.3%)	45 (61.6%)	
Necrosis (n = 171)				
Absent	61	43 (43.9%)	18 (24.6%)	0.008 *
Present/Focal	49	29 (29.6%)	20 (27.4%)	
Present/Comedo	61	26 (26.5%)	35 (48.0%)	
Margin status for DCIS (n = 171)				
Present with 2 mm	38	14 (14.3%)	24 (32.9%)	0.004 *
More than 2 mm away	133	84 (85.7%)	49 (67.1%)	
DCIS grade (n = 166)				
1	22	18 (19.3%)	4 (5.5%)	0.006 *
2	102	58 (62.4%)	44 (60.3%)	
3	42	17 (18.3%)	25 (34.2%)	
Invasion (n = 171)				
Absent	65	35 (35.7%)	30 (41.1%)	0.566
Present	106	63 (64.3%)	43 (58.9%)	
Invasive component type (n = 106)				
IC, NST	98	61 (96.8%)	37 (86%)	0.150
ILC	1	1 (1.6%)	0 (0%)	
TC	3	0 (0%)	3 (6.8%)	
IMC	3	1 (1.6%)	2 (4.6%)	
ILC AND TC	1	0 (0%)	1 (2.3%)	
Invasive component grade (n = 103)				
1	26	16 (26.2%)	10 (23.8%)	0.615
2	66	40 (64.6%)	26 (61.9%)	
3	11	5 (8.2%)	6 (14.3%)	
Margin status for invasive component (n = 106)				
Negative	103	63 (100%)	40 (93.0%)	0.083
Positive	3	0 (0.0%)	3 (7.0%)	
pT (n = 171)				
pTis	65	35 (35.7%)	30 (41.1%)	0.522
pT1mi	4	2 (2.0%)	2 (2.7%)	
pT1a	12	6 (6.1%)	6 (8.2%)	
pT1b	35	21 (21.4%)	14 (19.2%)	
pT1c	33	22 (22.4%)	11 (15.1%)	
pT2	18	10 (10.2%)	8 (10.9%)	
pT3	2	0 (0.0%)	2 (2.7%)	
pT4a	0	0 (0.0%)	0 (0.0%)	
pT4b	2	2 (2.0%)	0 (0.0%)	
pN (n = 171)				
pNx	55	34 (34.7%)	21 (28.7%)	0.801
pN0	88	49 (50.0%)	39 (53.4%)	
pN0 (i+)	1	1 (1.0%)	0 (0.0%)	
pN1a	14	7 (7.1%)	7 (9.6%)	
pN1mi	9	4 (4.0%)	5 (6.8%)	
pN2a	3	2 (2.0%)	1 (1.4%)	
pN3a	1	1 (1.0%)	0 (0.0%)	

Abbreviations: EIC: Extensive intraductal component; COL: Cancerization of lobules; DCIS: Ductal carcinoma in site; IC, NST: Invasive carcinoma, no special type; ILC: Invasive lobular carcinoma; TC: Tubular carcinoma; IMC: Invasive mucinous carcinoma. * Statistically significant (*p* < 0.05).

**Table 2:** Effect of presence of COL (COL-YES) on mastectomy pathological outcomes (using univariate analysis).

Clinicopathological Features	*p*-Value	OR
EIC	<0.001 *	11.20
% of blocks/slides with DCIS	<0.001 *	8.89
Necrosis, focal	0.22	1.65
Necrosis, comedo	0.002 *	3.26
Margin status for DCIS	0.006 *	2.94
DCIS grade	0.002 *	N/A
Invasion	0.47	0.80
Tumor stage	0.57	N/A
Lymph node status	0.63	1.26

* Statistically significant results (*p* < 0.05).

**Table 3:** Effect of presence of COL (COL-YES) on EIC, margin status, %of blocks/slides with DCIS, presence of comedo necrosis, DCIS grade (using multivariate analysis).

Clinicopathological Features	EIC (*p*-Value, OR *)	Margin Status for DCIS (*p*-Value, OR *)	% of Blocks/Sides with DCIS (*p*-Value)	Necrosis, Comedo (*p*-Value, OR *)	DCIS Grade (*p*-Value)
COL	0.002, 9.28	0.54	<0.001	0.17	0.01
DCIS Morphology					
Cribriform	0.64	0.34	0.07	0.008, 6.96	0.004
Solid	0.07	0.26	0.03	0.019, 5.54	0.59
Comedo	0.31	0.23	0.01	<0.001, 18.44	0.001
Papillary	0.84	0.23	0.06	0.15	0.28
Micropapillary	0.33	0.96	0.004	0.22	0.46
Necrosis	0.03, 4.73	0.11	0.34	N/A	<0.001
% Slides with DCIS	0.06	0.08	N/A	0.95	0.57
Patient Age	0.39	0.94	0.96	0.30	0.31
Invasion	N/A	0.25	0.04	0.50	0.64
Tumor Stage	0.23	0.75	0.96	0.49	0.61
Lymph Nodes Status	0.014, 6.06	0.08	0.52	0.14	0.53
Margin Status for DCIS	0.042	N/A	0.09	0.65	0.58
EIC	N/A	0.02, 5.38	0.05	0.09	0.46
DCIS Grade	0.79	0.55	0.58	0.13	N/A

* Included in the multivariate analysis for statistically significant results (*p* < 0.05).

**Table 4:** Effect of different pathological parameters on the presence of COL.

Clinicopathological Features	*p*-Value	OR
DCIS Morphology		
Cribriform	0.87	0.93
Solid	0.38	0.65
Comedo	0.007 *	0.15
Papillary	0.12	0.34
Micropapillary	0.21	0.46
Necrosis	0.83	1.07
% Slides with DCIS	<0.001 *	>99
Patient Age	0.07	0.97
Invasion	0.63	0.65
Tumor Stage	0.45	0.84
Lymph Nodes Status	0.73	1.28
Margin Status for DCIS	0.35	1.64
EIC	0.001 *	9.69
DCIS Grade	0.02 *	2.64

* Statistically significant results (*p* < 0.05).

## Data Availability

No new data were created or analyzed in this study. The original contributions presented in the study are included in the article, further inquiries can be directed to the corresponding author.
